# Effects of Selective Dorsal Rhizotomy on Ankle Joint Function in Patients With Cerebral Palsy

**DOI:** 10.3389/fped.2020.00075

**Published:** 2020-02-28

**Authors:** Filiz Ates, Joline E. Brandenburg, Kenton R. Kaufman

**Affiliations:** ^1^Motion Analysis Laboratory, Department of Orthopedic Surgery, Mayo Clinic, Rochester, MN, United States; ^2^Department of Physical Medicine and Rehabilitation, Mayo Clinic, Rochester, MN, United States; ^3^Department of Pediatrics and Adolescent Medicine, Mayo Clinic, Rochester, MN, United States; ^4^Department of Neurology, Mayo Clinic, Rochester, MN, United States

**Keywords:** cerebral palsy, joint quasi-stiffness, gait analysis, dynamic EMG, muscle contracture, ankle joint range of motion

## Abstract

Selective dorsal rhizotomy (SDR) is a neurosurgical technique performed to reduce muscle spasticity and improve motor functions in children with cerebral palsy (CP). In long term, muscle contractures were observed even after SDR. To better understand what is contributing to contracture formation, it is necessary to assess the effects of SDR on joint stiffness. We hypothesized that ankle passive range of motion (ROM) increases and the quasi-stiffness of the ankle joint decreases after SDR in children with CP. This retrospective study included 10 children with diplegic CP (median age 6 years 2 months) who had undergone SDR and for whom gait analysis data were collected 3 months before (Pre-SDR) and 13 months after (Post-SDR) surgery. Additional to clinical measures, ankle quasi-stiffness (the slope of the ankle moment vs. ankle angle plot) was analyzed from gait data. Passive ankle ROM at 0° (*p* < 0.0001) and 90° knee angles (*p* < 0.0001) increased after SDR. Dynamic EMG analysis showed improved phasic gastrocnemius activity (*p* < 0.0001). Equinus gait was improved with the reduction of peak plantar flexion (*p* < 0.0001), as well as an increase in peak dorsiflexion (*p* = 0.006) during walking was observed. Ankle joint quasi-stiffness (Pre- and post-SDR median = 0.056 Nm/kg/° and 0.051 Nm/kg/°, and interquartile range: 0.031 Nm/kg/° and 0.019 Nm/kg/°, respectively) decreased significantly (*p* = 0.0017) after SDR. Moreover, even though the total time of the gait cycle did not change (*p* = 0.99), the time interval from maximum dorsiflexion to maximum plantar flexion (Pre- and post-SDR median = 0.125 s and 0.156 s, and interquartile range: 0.153 and 0.253 s, respectively) increased significantly (*p* = 0.0068) after SDR. In conclusion, the decreased ankle quasi-stiffness and the enhanced time interval in the gait cycle due to SDR indicate better motor control and joint stability. Our findings suggest that the long-term contracture formation occurring even after surgical interventions may be related to the stiffening of non-contractile structures.

## Introduction

Selective dorsal rhizotomy (SDR) is a well-established neurosurgical technique performed to reduce muscle spasticity and improve motor functions in children with cerebral palsy (CP) (1–3). During SDR, nerve rootlets in the lower spine causing abnormal reflex circuits, are selectively cut under intraoperative neurophysiological guidance (4). Combined with physiotherapy, SDR reduces pain (5), improves joint range of motion (ROM) (6, 7), and enhances the gait of children with CP such that improvements in mobility are sustained for many years (6, 8–10). However, since more than half of the patients have additional orthopedic surgeries during these years (11, 12), isolation of the effects of SDR on long-term functional improvements is challenging.

Patients with CP develop muscle contractures that are thought to be multifactorial, including chronic muscle shortening (13) and decreased joint ROM (14) resulting in increased joint stiffness (15). The determinants of joint stiffness are (i) the passive component defined by the changes of the material properties of muscles and connective tissues, and (ii) the active component, which is the stiffness during dynamic conditions due to neural impairments (i.e., chronic muscle overactivity or spasticity) (16). Spasticity is believed to contribute to abnormal joint stiffness via changes of not only active but also passive muscle components due to adaptation (17). When the active component is temporarily reduced, such as through use of botulinum toxin, muscle stiffness may be slightly improved (18) but passive joint stiffness appears to be unchanged (19). Recent studies on animal muscles reported increased passive muscle forces and a higher amount of intramuscular collagenous tissues indicating enhanced passive stiffness after botulinum toxin administration (20–22). SDR, on the other hand, has the potential to improve joint stiffness since it permanently reduces or eliminates spasticity, thus reducing the abnormal active component without the use of botulinum toxins. However, there is no previous report on the effects of SDR on ankle joint stiffness. Understanding the effects of SDR on joint stiffness is particularly timely since it was recently argued that contracture formation occurs in long term SDR follow-up (5, 23). Therefore, it is useful to assess the effects of SDR on joint stiffness in order to better understand what is contributing to muscle contracture formation after SDR. The aim of this study was to test the hypotheses that (i) passive ankle ROM increases and (ii) ankle joint quasi-stiffness decreases during self-paced walking after SDR in children with CP.

## Methods

The present study was approved by the Institutional Review Board of Mayo Clinic, Rochester, MN. A retrospective medical record review was performed to identify children (18 years of age or younger) with a diagnosis of spastic diplegic CP who had undergone SDR. Children were included if they had received gait analysis evaluations within 18 months before (Pre-SDR) and 18 months after (Post-SDR) surgery. Children were excluded from the study if ankle kinetic or dynamic EMG data were missing.

### SDR Operation

One to two-level laminectomy procedures were applied to patients. Electrical stimulation was used to identify sensory roots from L2-S1 levels on both the left and right sides. The sensory roots at these levels were then separated into rootlets, with the number of rootlets sectioned per level varying depending on the electrophysiological response. In general, a sensory nerve was separated into 3–10 rootlets with one-third to three-fourths of the rootlets sectioned per side at each level. Taking into account all the sensory rootlets stimulated from L2-S1 levels, ~40–60% of rootlets were sectioned.

### Clinical Assessments

The Gross Motor Functional Classification System (GMFCS) (24) was used to classify the mobility of the patients.

### 3D Gait Analysis

A set of 3D markers was placed on the body of each subject, including the sacrum and bilaterally on the acromion processes, lateral epicondyle of the elbows, center of the dorsum of the wrists, anterior superior iliac spines (ASIS), lateral femoral condyles, lateral malleoli, mid-thigh, mid-shank, heels, and the spaces between the first and second metatarsal heads. The model used was the same as described by Kadaba et al. (25) except for the foot. A three-dimensional coordinate system was used for the foot with an anteriorly directed x-axis, y-axis pointing to the body's left side, and a superiorly directed z-axis. The markers placed at bony prominences were used for establishing anatomic coordinate systems for the pelvis, thigh, shank, and foot. Additional tracking markers were applied to each segment. Two additional markers bilaterally on the medial femoral condyle and the medial malleoli were used to locate the joint centers.

For gait analysis, a 10-camera motion capture system (Raptor 12, MotionAnalysis, Santa Rosa, CA, USA) was used. The 3D coordinates of the markers and force plate were used as inputs to a commercial software program (Visual3D, C-Motion Inc., Rockville, MD), to calculate the joint kinematics and kinetics. The Visual3D program was used to define the joint center and segment coordinate systems from the 3D marker trajectories, as well as the subsequent rigid body kinematic/kinetic calculations. Embedded right-hand Cartesian coordinate systems were used in this model to describe the position and orientation of the rigid body segments of the lower extremity. A three-dimensional coordinate system was used for the foot with an anteriorly directed x-axis, y-axis pointing to the body's left side, and a superiorly directed z-axis. With these embedded coordinate systems, the joint angles were determined using the floating-axis or Euler angle convention. The ankle center was located by a vector directed medially from the lateral malleolus marker at one-half the distance of the measured ankle width. The segmental joint forces and moments were derived based on the calculated joint centers.

### EMG

A 16 channel surface EMG system (MA300, Motion Lab Systems, Inc., Baton Rouge, LA) was used for data collection and synchronized with the kinematic data. EMG data were collected at 2,400 Hz from the rectus femoris, hamstrings, tibialis anterior, peroneus longus, and gastrocnemius muscles rectified and passed through a fourth-order low pass Butterworth filter with 6 Hz cutoff using custom Matlab (Mathworks, Natick, MA) programming. Placement of EMG electrodes is confirmed by real-time visual observation of the EMG signal while muscle activation is achieved through resisted motion.

### Assessment of Passive Characteristics

Ankle passive ROM was recorded at two knee angles: (i) The patient was positioned supine with the knee fully extended (at 0°) and (ii) the patient was positioned prone with the knee at 90°. The foot was positioned in neutral inversion and eversion. The proximal arm of the goniometer was aligned with the lateral midline of the lower leg using the head of the fibula as a reference point. By aligning the distal arm of the goniometer parallel to the fifth metatarsal, the examiner changed the ankle angle slowly to measure max PF and max DF.

### Assessments of Dynamic Characteristics

#### Maximum Plantar Flexion (PF) and Dorsiflexion (DF) Angles During Walking

To quantify the effects of SDR on the ankle angle used during walking, the maximum PF and DF angles were calculated from the average of three trials for each participant.

#### Ankle Joint Quasi-Stiffness

**Figure 1:**
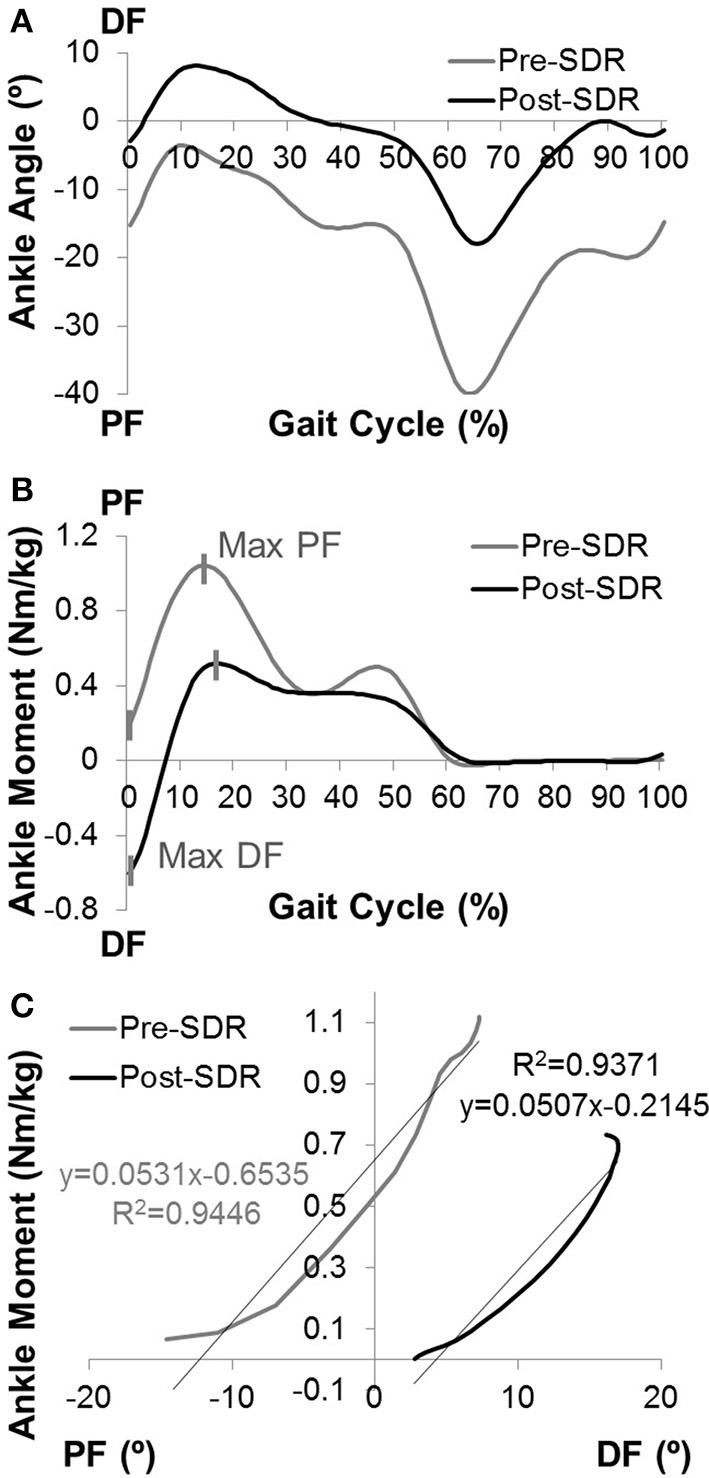
The computation of ankle joint quasi-stiffness. Examples of **(A)** ankle angle vs. gait cycle and **(B)** ankle moment vs. gait cycle data for pre- and post-SDR. **(C)** The ankle angle vs. ankle moment data is shown for the chosen time interval from max DF to max PF for post-SDR. Since some of the children did not have DF moment before the surgery, the time interval was chosen from min PF to max PF. The quasi-stiffness is defined as the slope of the linear regression curve for this time interval.

The Visual3D computer software package along with a set of custom Matlab (Mathworks, Natick, MA) programs were used for data reduction and database archival. Three trials were collected from each participant. The period of a gait cycle (stride) was defined from the initial contact of one lower extremity to the next initial contact of that same extremity. Kinematic measurements included the motion of the body and limb segments during representative walking strides. The kinetic analysis included the net forces and torques (moment) exerted on the body as a result of the combined effects of the ground reaction force, inertia, and muscle contraction. The joint moments were described using an internal moment convention. 3D kinematics was reported in degrees, and kinetics was normalized to body mass. Figure 1 shows examples of (A) ankle angle and (B) ankle moment plotted as functions of the gait cycle.

By using raw time-series kinematics and kinetics, the ankle moment was calculated as a function of ankle PF-DF angles for each trial. During the first max PF in early stance to max DF in midstance (second rocker interval); the shank moving forward is controlled by the eccentric contraction of the gastrocnemius and soleus. Davis and DeLuca (26) described the slope of the ankle moment—ankle angle plot at the second rocker interval of the gait as dynamic ankle joint stiffness for healthy participants. However, most of the children with CP do not have a traditional second rocker interval. They tend to have premature rise to peak PF moment. For this reason, the present study utilizes the ankle moment-gait cycle relationship to determine the time interval from max DF moment (min PF moment if there is no DF moment) to max PF moment (Figure 1B).

Moreover, as there has been concerns raised regarding the precision of the term “dynamic stiffness” in this context, previous literature (27) introduced the term “quasi-stiffness” to define the resistance to angular motion.

The slope of the moment (Nm/kg) vs. ankle angle (degrees) at this specific time interval was determined using a linear regression model (Figure 1C). The Pearson correlation coefficient (r) for each trial was calculated. If the correlation was high (*r* ≥ 0.80), the calculated slope was recorded as ankle joint quasi-stiffness. If high correlations were observed for more than one trial for each limb, the average of the slopes of trials was recorded as quasi-stiffness.

#### Dynamic EMG

**Figure 2:**
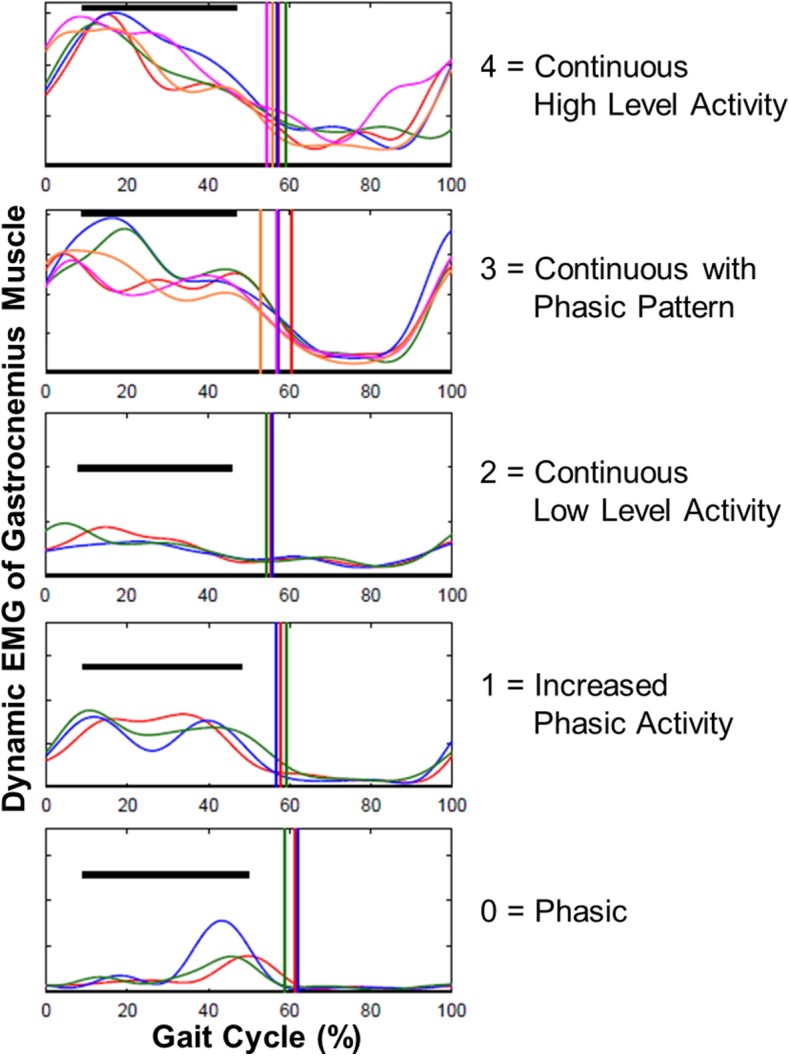
Examples for scoring the gastrocnemius muscle EMG data with respect to gait cycle. The phasic character of gastrocnemius was scored as: 0, Phasic; 1, Increased Phasic Activity; 2, Continuous Low Level Activity; 3, Continuous with Phasic Pattern; 4, Continuous High Level Activity.

Enhanced gastrocnemius muscle tone is defined as one of the major determinants of ankle joint stiffness. Therefore, the phasic character of the gastrocnemius muscle was scored by using dynamic EMG data with respect to the gait cycle: 0, Phasic; 1, Increased Phasic Activity; 2, Continuous Low-Level Activity; 3, Continuous with Phasic Pattern; 4, Continuous High-Level Activity (Figure 2). Scoring was performed by one examiner and intra-rater reliability was assessed with Cohen's kappa (κ).

### Statistical Analyses

For statistical analyses, SAS 9.4 (SAS Institute, Cary, NC) software was used. To take into account that right and left limbs may provide correlated response data, generalized estimating equations were used separately to evaluate if significant changes occurred in (i) ankle joint passive ROM, (ii) dynamic EMG scores, (iii) max PF and DF angles during walking, (iv) joint quasi-stiffness, (v) the time interval from max DF to max PF, and (vi) total gait cycle time between Pre-SDR and Post-SDR. To test the correlation between the effects of SDR on passive and dynamic parameters, Spearman's rank correlation coefficient (*r*_s_) was calculated between the absolute change in ankle passive ROM and quasi-stiffness due to SDR. Differences were considered significant if *p* < 0.05.

## Results

A total of 10 children with spastic diplegic CP underwent SDR and had pre- and post-SDR gait analysis. There were five male and five female children (median age 6 years 2 month at the time of surgery; min: 3 years 6 months, max: 8 years 7 months, interquartile range: 2 years 8 months). Pre-SDR and post-SDR clinical tests and gait analyses were performed 3 months (min: 1 month, max: 8 months, interquartile range: 3 months and 21 days) before surgery and 13 months (min: 10 months, max: 17 months, interquartile range: 3 months and 9 days) after surgery, respectively. Two children were operated on unilaterally. The data collected from their non-operated legs were excluded from the analyses. Pre-SDR GMFCS levels ranged from I to III. GMFCS levels of half of the children improved post-SDR (Table 1).

**Table 1:** Patient mobility, dynamic EMG Scores, and ankle characteristics during walking.

**#**	**Age**	**Sex**	**GMFCS**		**EMG-L**		**EMG-R**		**Max PF-L**		**Max PF-R**		**Max DF-L**		**Max DF-R**	
			**Pre**	**Post**	**Pre**	**Post**	**Pre**	**Post**	**Pre**	**Post**	**Pre**	**Post**	**Pre**	**Post**	**Pre**	**Post**
1	7 (8)	M	I	I	4	1	4	1	−40.2	−18.0	−30.1	−12.4	−6.5	8.1	6.9	8.9
2	8 (7)	M	II	II	2	1	1	0	−19.1	−11.9	−11.4	−2.3	3.9	9.1	8.9	14.0
3	7 (3)	M	II	II	1	0	2	0	−32.8	−12.8	−21.8	−26.1	14.1	19.3	10.3	19.2
4	6 (2)	M	III	II	3	0	2	1	−6.3	−12.3	−3.2	−1.4	13.0	12.6	7.4	12.2
5	5 (5)	F	II	I	3	1	3	1	−21.4	−19.4	−60.3	−14.5	12.4	21.0	−4.9	20.1
6	3 (8)	F	III	III	3	0	3	0	−50.4	−55.0	−21.6	−37.1	−3.4	3.6	13.7	5.8
7	5 (0)	F	III	II	1	1	2	1	−49.0	−15.7	−55.2	−29.0	−24.2	11.7	−33.8	4.8
8	6 |(2)	F	II	I	1	3	1	0	−40.1	−13.4	−29.0	−10.9	−10.5	6.0	4.0	15.2
9	3 (6)	F	I	I	3	1	2	0	−31.8	−11.8	−32.8	−17.3	5.3	−16.4	13.0	16.7
10	7 (4)	M	II	I	2	2	2	2	1.1	−3.4	4.2	−7.9	12.6	19.1	11.7	8.1

*Age is presented as year (month). M, F, R, L refer to male, female, right limb, and left limb. Pre and Post-refer to Pre-SDR and Post-SDR conditions. EMG grades 0, Phasic; 1, Increased Phasic Activity; 2, Continuous Low Level Activity; 3, Continuous with Phasic Pattern; 4, Continuous High Level Activity*.

**Figure 3:**
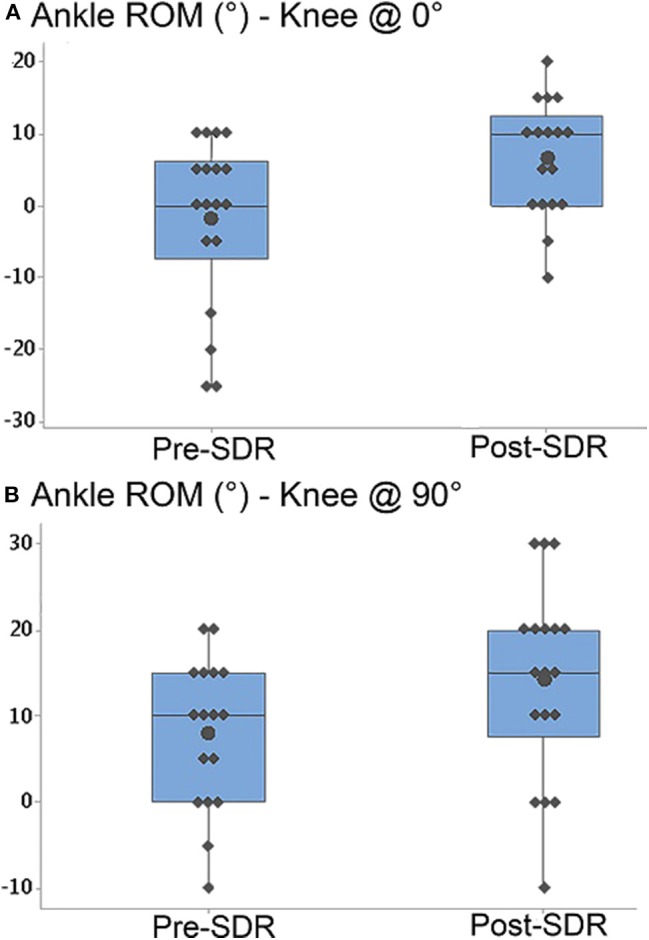
Box-and-whisker plot of passive ankle range of motion (ROM) **(A)** when the knee was at 0° (fully extended) and **(B)** when the knee was at 90° for pre- and post-SDR. Negative and positive ankle ROM values indicate plantar flexion and dorsiflexion positions, respectively. Pre- and Post-SDR values are significantly different for both A and B.

Ankle joint passive ROM measured at 0° of knee angle (*p* < 0.0001) (pre- and post-SDR median = 0 and 10°, interquartile range: 13.75 and 12.5°, respectively; Figure 3A) and at 90° of knee angle (*p* < 0.0001) (pre- and post-SDR median = 10° and 15°, interquartile range: 15 and 12.5°, respectively; Figure 3B) increased significantly due to SDR.

After SDR, max PF during walking decreased significantly (*p* < 0.0001) (pre- and post-SDR median = −29.54 and −13.10°, interquartile range: 26.89 and 7.90°, respectively) and max DF increased significantly (*p* < 0.0001) (pre- and post-SDR median = 7.15 and 11.93°, interquartile range: 17.04 and 11.95°, respectively).

Dynamic EMG analysis showed that phasic pattern scores of gastrocnemius significantly improved after SDR (*p* < 0.0001; Table 1). Intra-rater reliability of scoring was moderate to high for both pre- (κ = 0.78) and post-SDR (κ = 0.72) assessments.

**Figure 4:**
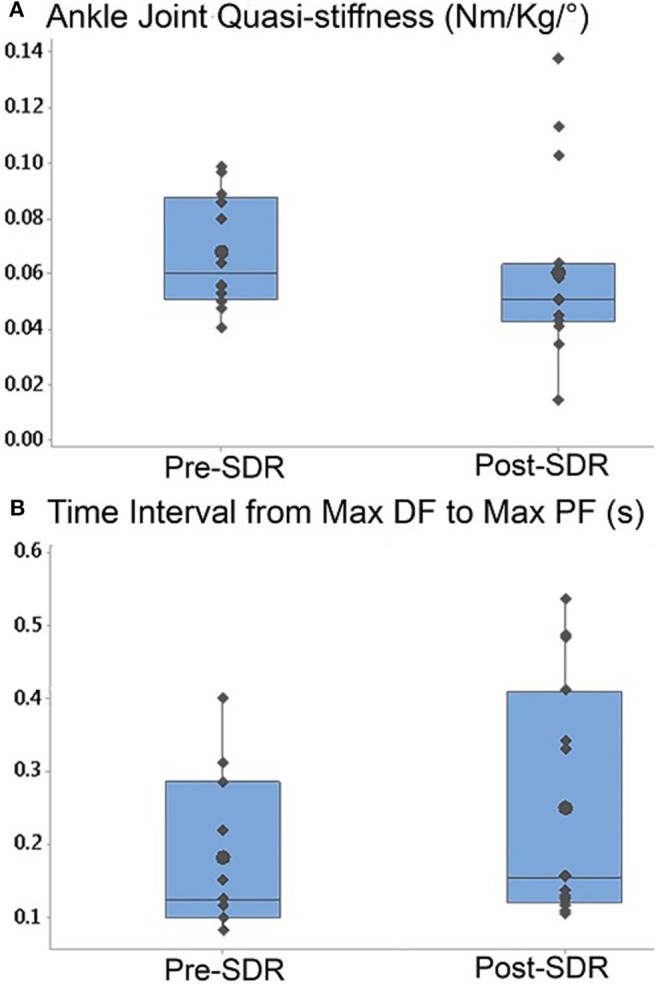
Box-and-whisker plot of **(A)** the ankle joint quasi-stiffness and **(B)** the time interval from max DF moment to max PF moment for Pre- and Post-SDR. Pre- and Post-SDR values are significantly different for both A and B.

Ankle joint quasi-stiffness (Pre- and post-SDR median = 0.056 Nm/kg/° and 0.051 Nm/kg/°, and interquartile range: 0.031 Nm/kg/° and 0.019 Nm/kg/°, respectively) decreased significantly (*p* = 0.0017) after SDR surgery (Figure 4A). Moreover, even though the total time of the gait cycle (pre- and post-SDR median = 0.868 and 0.935 s, and interquartile range: 1.308 and 0.284 s, respectively) did not change (*p* = 0.99), the time interval from max DF to max PF (pre- and post-SDR median = 0.125 and 0.156 s, and interquartile range: 0.153 and 0.253 s, respectively) significantly increased (*p* = 0.0068) after SDR surgery (Figure 4B).

No significant correlation was found between the changes in ankle passive ROM and the changes in quasi-stiffness [*r*_s_ = 0.36 for ankle ROM at 0° knee angle vs. stiffness (*p* = 0.30) and *r*_s_ = 0.24 for ankle ROM at 90° knee angle vs. stiffness (*p* = 0.41)].

## Discussion

The present study showed increased passive ankle joint ROM and decreased quasi-stiffness for children with CP after SDR. These results support both of our hypotheses. Furthermore, we found that the time interval from max DF to max PF during gait was elevated substantially by SDR. Numerous studies (3, 5, 6, 11) have reported motor function improvement for children with CP after SDR. Together with the other passive and active outcome measures presenting improvement, the decreased quasi-stiffness and the increased time interval might be associated with better motor control and an indication of enhanced joint stability post-SDR.

Increased passive ROM of ankle joint both at knee flexion and extension indicates improvement in passive mechanical characteristics of all plantar flexor muscles. Additionally, gastrocnemius EMG revealed an improved phasic activity after SDR. This also contributed to a reduction in passive joint stiffness. As the children with CP underwent physical therapy (per our program's post-SDR therapy protocol), the improvement in passive ROM may be more associated with the physical therapy intervention, than SDR alone. A previous study (9) showed that the improvement in passive joint stiffness observed a year after surgery was sustained even 20 years after SDR. This emphasizes the major progressive role of SDR. Nevertheless, together with the physical therapy, the entire effect of SDR on the passive ankle joint ROM is substantial.

We found that the dynamic gait characteristics of the ankle joint improve after SDR. Specifically, a significant increase in max DF and reduction of max PF indicate a substantial shift of ankle angles used during walking. The shift in ankle angles suggests a reduction in equinus gait after SDR in children with CP, which is consistent with previous gait analysis data (28, 29). Thus, gait in children with CP who undergo SDR appears to shift the gait kinematics toward a more typically developing gait, potentially due to changes in motor control.

On the other hand, the changes in passive ankle ROM do not correlate with the joint quasi-stiffness. This could be due to the fact that the passive behavior of joints comprises not only the passive muscle characteristics but also the amount and the stiffness of non-muscular structures such as fascial connective tissues, aponeurosis, ligaments, and nerves (30, 31). Therefore, this finding suggests the need for new studies on the active and passive characteristics of individual muscles as well as stiffness of non-muscular structures. Individual muscle stiffness in relation to joint stiffness can be identified by using e.g., ultrasound elastography method. A previous study (32) using the shear wave elastography (SWE) approach reported that the gastrocnemius in its passive state is stiffer in children with CP than in children with typical development. The SWE approach can be reliably used [e.g., (33)] to evaluate the stiffness of muscles activated at different intensities up to maximum voluntary contraction for healthy adults. Further studies evaluating the correlation between passive muscle properties and stiffness of individual muscles during dynamic conditions in children with CP and the effects of SDR on these properties should be considered.

Decreased joint ROM and increased joint stiffness, which characterize spastic CP are associated with changes in the material properties of contractile and non-contractile structures (14). Repetitive spastic contractions that cause muscle shortening (13) are typically accepted as the main reason for contracture formation of the muscles crossing the related joint. It has also been reported, by using an instrumented and model-based approach that the active neural contribution of plantar flexor muscles is a major source of higher ankle joint stiffness in CP (16). In line with these previous works, our study demonstrates that SDR diminishes the neural source of spasticity and improves ankle joint quasi-stiffness. However, Tedroff et al. argued that anti-spasticity treatment may not prevent long-term muscle contractures (5). Consistent with this argument, Olsson et al. (34) reported that increased passive tension *in vivo* is unrelated to the stretch reflex for spastic vastus lateralis of spinal cord injured patients by using EMG and dynamometer data collected during knee flexion and extension. From the biopsied muscle samples, they showed fiber type alterations and higher passive tension at the cell level due to spasticity as well (34). Moreover, using hamstring biopsies Smith et al. (35) showed that not muscle fibers but fiber bundles comprising the extracellular matrix of children with CP were stiffer than that of age-matched controls. Recent studies on active mechanical characteristics of muscles of children with CP (36–38) indicated that fascial structures have a prominent role during co-activity of synergistic and antagonistic muscles. Therefore, passive structures such as connective tissues can modify the active force production capacity of muscles in CP. Together with these previous reports, our findings suggest that further research aiming to understand muscle contracture formation should focus on mechanical alterations of non-contractile structures and their specific effects on active muscle force production.

It should be noted that our study is limited with 10 patients with a comparably short follow-up period since the operation has not been performed in our clinic for long. Moreover, clinical evaluations of the strength of joints involved and selective control are important for understanding the long-term stability of the joints. However, these evaluations were not completed and reported for the patients included in this study. Post-SDR ankle contractures occur later stages or after childhood and adolescent growth. Therefore, to better understand the pathophysiology of post-SDR ankle contractures, the relationship between ankle weakness, motor control, and ankle joint stiffness, including ankle quasi-stiffness analysis will be necessary in long-term follow-up studies. Further, for dynamic activities, investigating stiffness is not only essential for the ankle but also the knee and the hip joints. Hence, in the future, one should also consider pre- and post-SDR quasi-stiffness calculations for the knee and hip joints in order to advance treatment strategies and prevent later contractures.

In conclusion, the present study shows that passive and dynamic ankle joint characteristics improve after SDR. Significant changes in ankle joint quasi-stiffness and in the time interval from max DF to max PF may indicate better motor control together with other outcomes, which reveal progress in dynamic joint parameters. Further research on muscle contracture following SDR is suggested, in particular with a focus on mechanical alterations of intramuscular non-contractile structures as well as non-muscular tissues.

## Data Availability Statement

The datasets generated for this study are available on request to the corresponding author.

## Ethics Statement

The studies involving human participants were reviewed and approved by Mayo Clinic Institutional Review Board (IRB) (#16-010165).

## Author Contributions

FA, JB, and KK contributed to the conception and design of the study as well as the interpretation of the results. FA performed data analysis and manuscript writing. KK contributed to writing, editing, and critical appraisal of the manuscript. All authors approved the final submitted manuscript.

### Conflict of Interest

The authors declare that the research was conducted in the absence of any commercial or financial relationships that could be construed as a potential conflict of interest.
